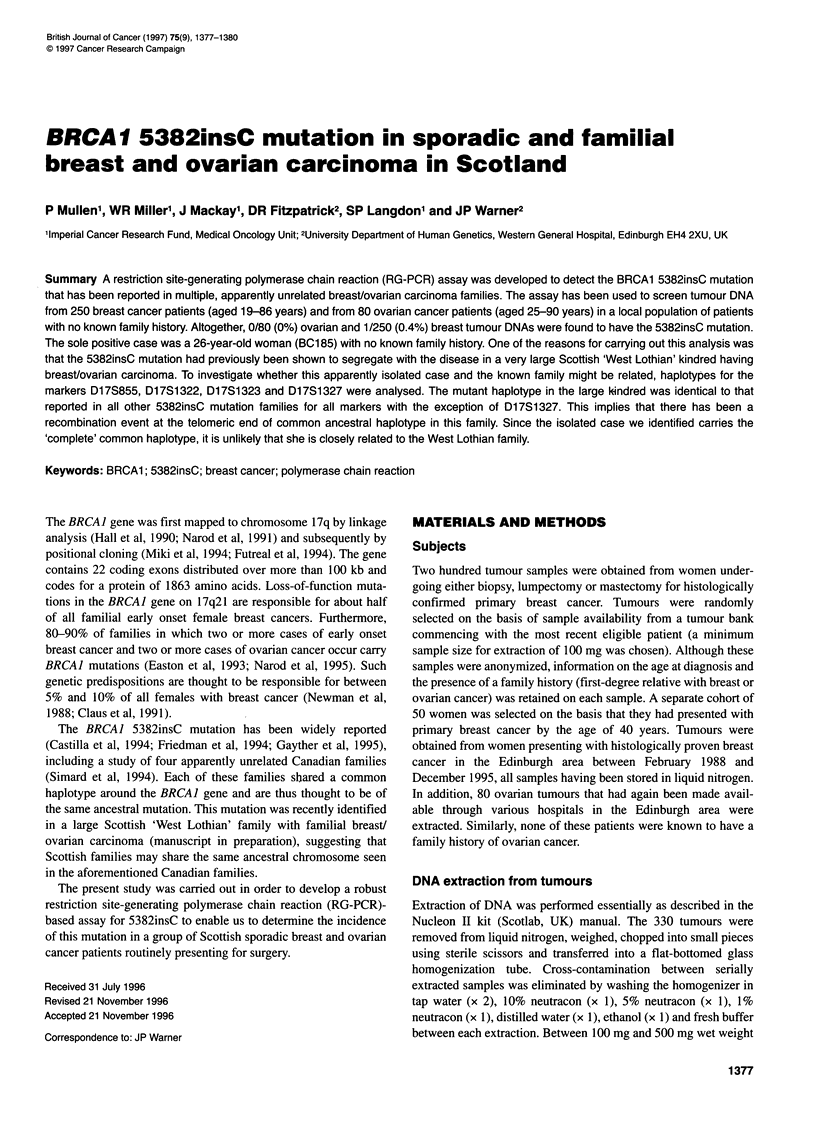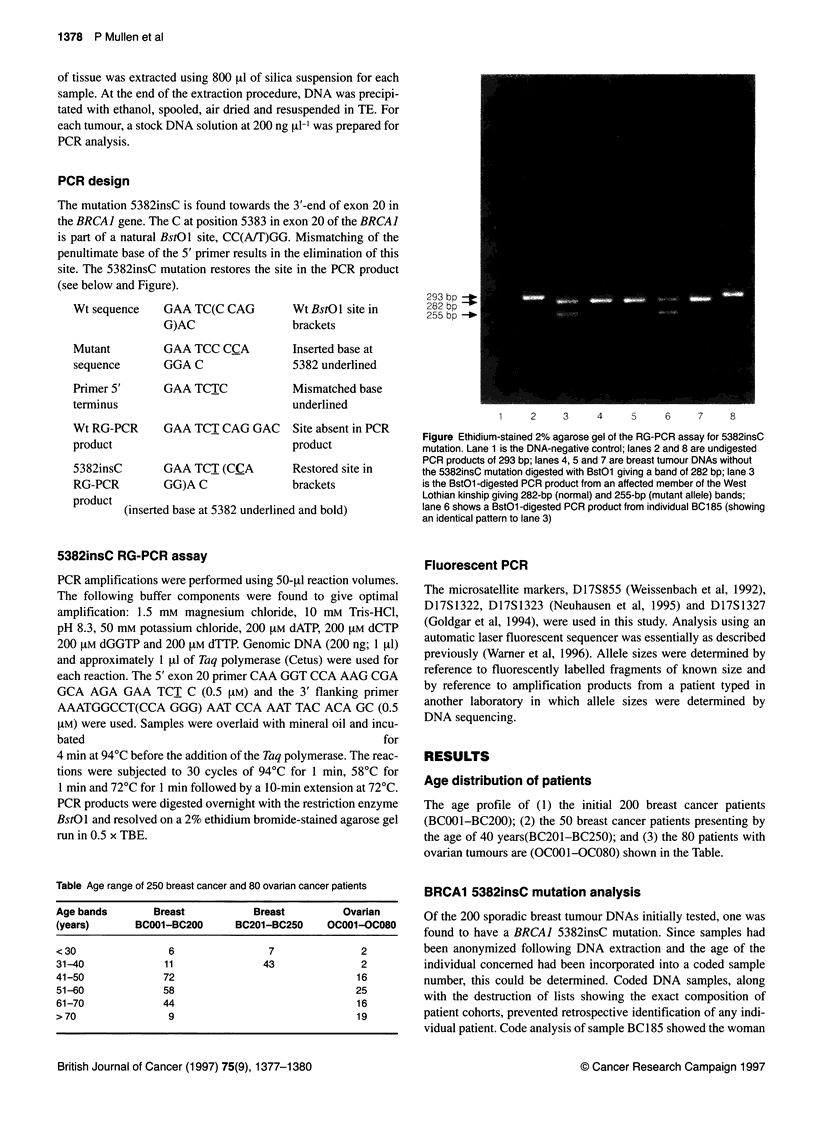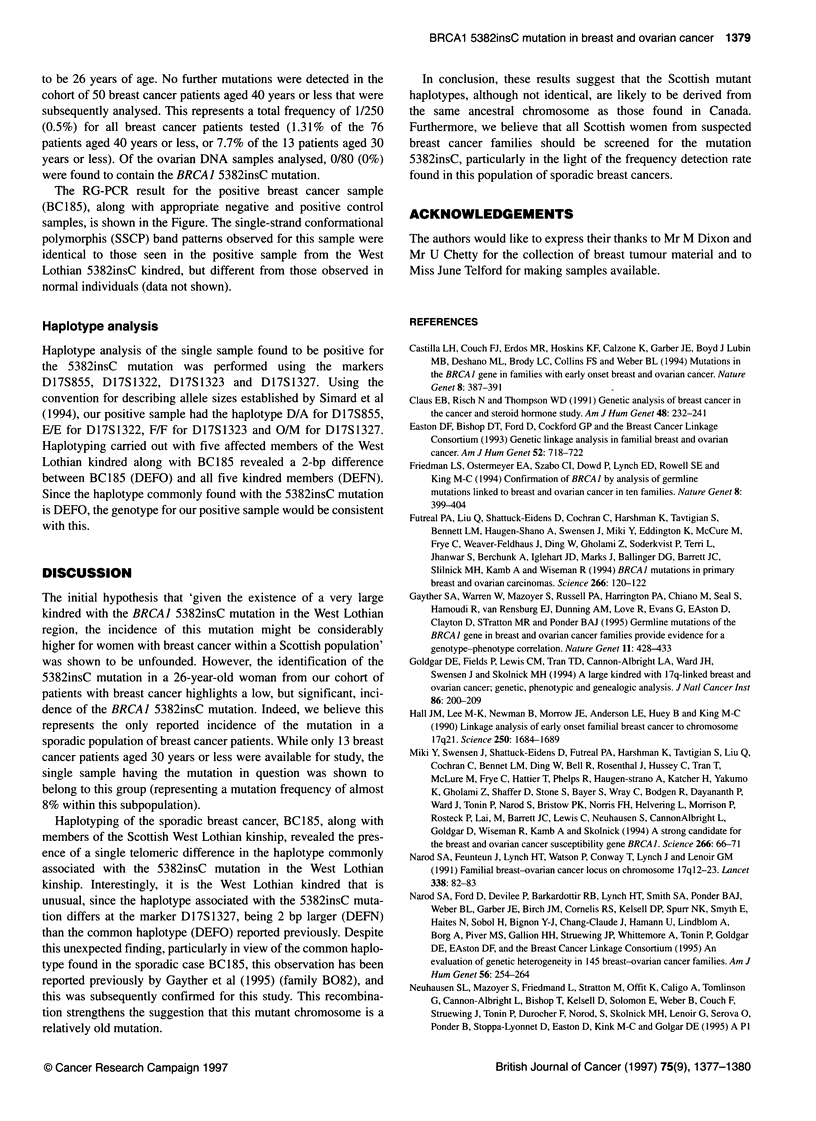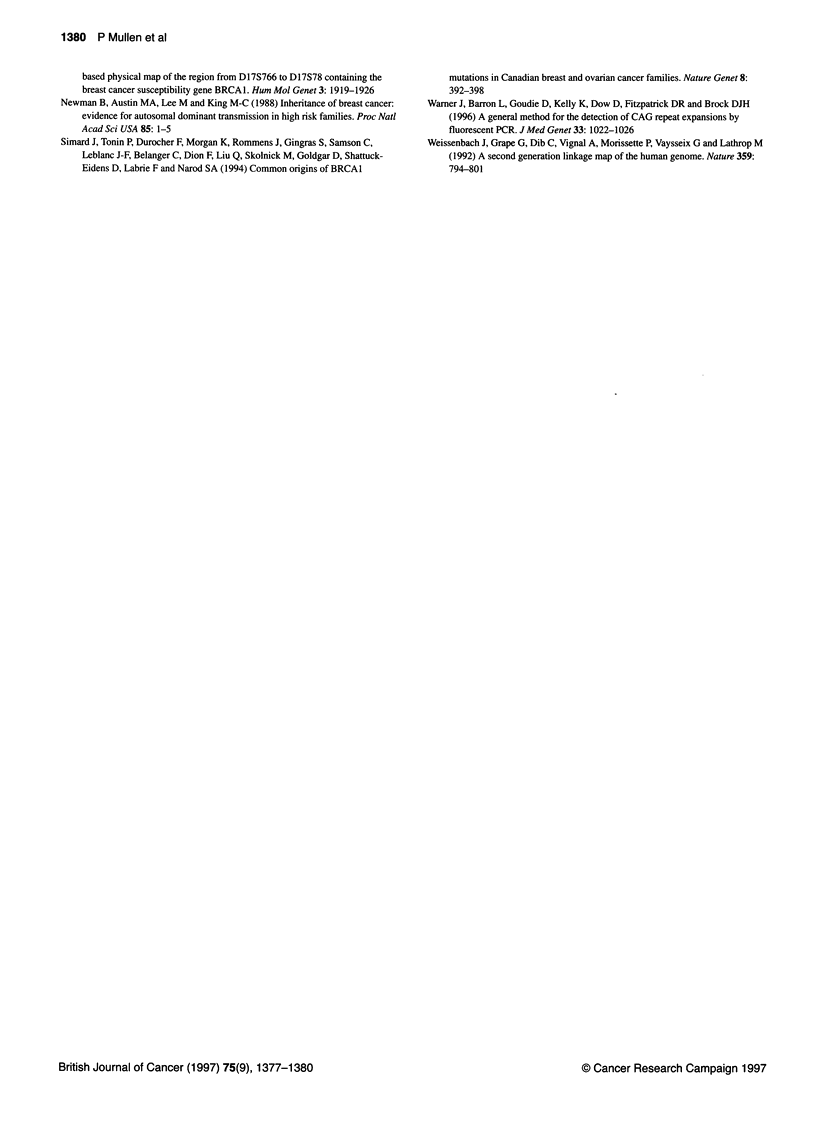# BRCA1 5382insC mutation in sporadic and familial breast and ovarian carcinoma in Scotland.

**DOI:** 10.1038/bjc.1997.233

**Published:** 1997

**Authors:** P. Mullen, W. R. Miller, J. Mackay, D. R. Fitzpatrick, S. P. Langdon, J. P. Warner

**Affiliations:** Imperial Cancer Research Fund, Medical Oncology Unit, Western General Hospital, Edinburgh, UK.

## Abstract

**Images:**


British Journal of Cancer (1997) 75(9), 1377–1380
© 1997 Cancer Research Campaign

### *BRCA1* 5382insC mutation in sporadic and familial breast and ovarian carcinoma in Scotland

**P Mullen[1], WR Miller[1], J Mackay[1], DR Fitzpatrick[2], SP Langdon[1] and JP Warner[2]**

[1]Imperial Cancer Research Fund, Medical Oncology Unit; [2]University Department of Human Genetics, Western General Hospital, Edinburgh EH4 2XU, UK

**Summary** A restriction site-generating polymerase chain reaction (RG-PCR) assay was developed to detect the BRCA1 5382insC mutation that has been reported in multiple, apparently unrelated breast/ovarian carcinoma families. The assay has been used to screen tumour DNA from 250 breast cancer patients (aged 19–86 years) and from 80 ovarian cancer patients (aged 25–90 years) in a local population of patients with no known family history. Altogether, 0/80 (0%) ovarian and 1/250 (0.4%) breast tumour DNAs were found to have the 5382insC mutation. The sole positive case was a 26-year-old woman (BC185) with no known family history. One of the reasons for carrying out this analysis was that the 5382insC mutation had previously been shown to segregate with the disease in a very large Scottish 'West Lothian' kindred having breast/ovarian carcinoma. To investigate whether this apparently isolated case and the known family might be related, haplotypes for the markers D17S855, D17S1322, D17S1323 and D17S1327 were analysed. The mutant haplotype in the large kindred was identical to that reported in all other 5382insC mutation families for all markers with the exception of D17S1327. This implies that there has been a recombination event at the telomeric end of common ancestral haplotype in this family. Since the isolated case we identified carries the 'complete' common haplotype, it is unlikely that she is closely related to the West Lothian family.

**Keywords:** BRCA1; 5382insC; breast cancer; polymerase chain reaction

The *BRCA1* gene was first mapped to chromosome 17q by linkage analysis (Hall et al, 1990; Narod et al, 1991) and subsequently by positional cloning (Miki et al, 1994; Futreal et al, 1994). The gene contains 22 coding exons distributed over more than 100 kb and codes for a protein of 1863 amino acids. Loss-of-function mutations in the *BRCA1* gene on 17q21 are responsible for about half of all familial early onset female breast cancers. Furthermore, 80–90% of families in which two or more cases of early onset breast cancer and two or more cases of ovarian cancer occur carry *BRCA1* mutations (Easton et al, 1993; Narod et al, 1995). Such genetic predispositions are thought to be responsible for between 5% and 10% of all females with breast cancer (Newman et al, 1988; Claus et al, 1991).

The *BRCA1* 5382insC mutation has been widely reported (Castilla et al, 1994; Friedman et al, 1994; Gayther et al, 1995), including a study of four apparently unrelated Canadian families (Simard et al, 1994). Each of these families shared a common haplotype around the *BRCA1* gene and are thus thought to be of the same ancestral mutation. This mutation was recently identified in a large Scottish 'West Lothian' family with familial breast/ovarian carcinoma (manuscript in preparation), suggesting that Scottish families may share the same ancestral chromosome seen in the aforementioned Canadian families.

The present study was carried out in order to develop a robust restriction site-generating polymerase chain reaction (RG-PCR)-based assay for 5382insC to enable us to determine the incidence of this mutation in a group of Scottish sporadic breast and ovarian cancer patients routinely presenting for surgery.

Received 31 July 1996
Revised 21 November 1996
Accepted 21 November 1996

Correspondence to: JP Warner

### MATERIALS AND METHODS

### Subjects

Two hundred tumour samples were obtained from women undergoing either biopsy, lumpectomy or mastectomy for histologically confirmed primary breast cancer. Tumours were randomly selected on the basis of sample availability from a tumour bank commencing with the most recent eligible patient (a minimum sample size for extraction of 100 mg was chosen). Although these samples were anonymized, information on the age at diagnosis and the presence of a family history (first-degree relative with breast or ovarian cancer) was retained on each sample. A separate cohort of 50 women was selected on the basis that they had presented with primary breast cancer by the age of 40 years. Tumours were obtained from women presenting with histologically proven breast cancer in the Edinburgh area between February 1988 and December 1995, all samples having been stored in liquid nitrogen. In addition, 80 ovarian tumours that had again been made available through various hospitals in the Edinburgh area were extracted. Similarly, none of these patients were known to have a family history of ovarian cancer.

### DNA extraction from tumours

Extraction of DNA was performed essentially as described in the Nucleon II kit (Scotlab, UK) manual. The 330 tumours were removed from liquid nitrogen, weighed, chopped into small pieces using sterile scissors and transferred into a flat-bottomed glass homogenization tube. Cross-contamination between serially extracted samples was eliminated by washing the homogenizer in tap water (× 2), 10% neutracon (× 1), 5% neutracon (× 1), 1% neutracon (× 1), distilled water (× 1), ethanol (× 1) and fresh buffer between each extraction. Between 100 mg and 500 mg wet weight of tissue was extracted using 800 μl of silica suspension for each sample. At the end of the extraction procedure, DNA was precipitated with ethanol, spooled, air dried and resuspended in TE. For each tumour, a stock DNA solution at 200 ng $\mu l^{-1}$ was prepared for PCR analysis.

### PCR design

The mutation 5382insC is found towards the 3′-end of exon 20 in the *BRCA1* gene. The C at position 5383 in exon 20 of the *BRCA1* is part of a natural *Bst*O1 site, CC(A/T)GG. Mismatching of the penultimate base of the 5′ primer results in the elimination of this site. The 5382insC mutation restores the site in the PCR product (see below and Figure).

| | | |
|---|---|---|
| Wt sequence | GAA TC(C CAG G)AC | Wt *Bst*O1 site in brackets |
| Mutant sequence | GAA TCC CCA GGA C | Inserted base at 5382 underlined |
| Primer 5′ terminus | GAA TCTC | Mismatched base underlined |
| Wt RG-PCR product | GAA TCT CAG GAC | Site absent in PCR product |
| 5382insC RG-PCR product | GAA TCT (CCA GG)A C | Restored site in brackets |

(inserted base at 5382 underlined and bold)

### 5382insC RG-PCR assay

PCR amplifications were performed using 50-μl reaction volumes. The following buffer components were found to give optimal amplification: 1.5 mM magnesium chloride, 10 mM Tris-HCl, pH 8.3, 50 mM potassium chloride, 200 μM dATP, 200 μM dCTP 200 μM dGGTP and 200 μM dTTP. Genomic DNA (200 ng; 1 μl) and approximately 1 μl of *Taq* polymerase (Cetus) were used for each reaction. The 5′ exon 20 primer CAA GGT CCA AAG CGA GCA AGA GAA TCT C (0.5 μM) and the 3′ flanking primer AAATGGCCT(CCA GGG) AAT CCA AAT TAC ACA GC (0.5 μM) were used. Samples were overlaid with mineral oil and incubated for 4 min at 94°C before the addition of the *Taq* polymerase. The reactions were subjected to 30 cycles of 94°C for 1 min, 58°C for 1 min and 72°C for 1 min followed by a 10-min extension at 72°C. PCR products were digested overnight with the restriction enzyme *Bst*O1 and resolved on a 2% ethidium bromide-stained agarose gel run in 0.5 × TBE.

**Table** Age range of 250 breast cancer and 80 ovarian cancer patients

| Age bands (years) | Breast BC001–BC200 | Breast BC201–BC250 | Ovarian OC001–OC080 |
|---|---|---|---|
| < 30 | 6 | 7 | 2 |
| 31–40 | 11 | 43 | 2 |
| 41–50 | 72 | | 16 |
| 51–60 | 58 | | 25 |
| 61–70 | 44 | | 16 |
| > 70 | 9 | | 19 |

**Figure** Ethidium-stained 2% agarose gel of the RG-PCR assay for 5382insC mutation. Lane 1 is the DNA-negative control; lanes 2 and 8 are undigested PCR products of 293 bp; lanes 4, 5 and 7 are breast tumour DNAs without the 5382insC mutation digested with BstO1 giving a band of 282 bp; lane 3 is the BstO1-digested PCR product from an affected member of the West Lothian kinship giving 282-bp (normal) and 255-bp (mutant allele) bands; lane 6 shows a BstO1-digested PCR product from individual BC185 (showing an identical pattern to lane 3)

### Fluorescent PCR

The microsatellite markers, D17S855 (Weissenbach et al, 1992), D17S1322, D17S1323 (Neuhausen et al, 1995) and D17S1327 (Goldgar et al, 1994), were used in this study. Analysis using an automatic laser fluorescent sequencer was essentially as described previously (Warner et al, 1996). Allele sizes were determined by reference to fluorescently labelled fragments of known size and by reference to amplification products from a patient typed in another laboratory in which allele sizes were determined by DNA sequencing.

### RESULTS

### Age distribution of patients

The age profile of (1) the initial 200 breast cancer patients (BC001–BC200); (2) the 50 breast cancer patients presenting by the age of 40 years(BC201–BC250); and (3) the 80 patients with ovarian tumours are (OC001–OC080) shown in the Table.

### BRCA1 5382insC mutation analysis

Of the 200 sporadic breast tumour DNAs initially tested, one was found to have a *BRCA1* 5382insC mutation. Since samples had been anonymized following DNA extraction and the age of the individual concerned had been incorporated into a coded sample number, this could be determined. Coded DNA samples, along with the destruction of lists showing the exact composition of patient cohorts, prevented retrospective identification of any individual patient. Code analysis of sample BC185 showed the woman

© Cancer Research Campaign 1997

to be 26 years of age. No further mutations were detected in the cohort of 50 breast cancer patients aged 40 years or less that were subsequently analysed. This represents a total frequency of 1/250 (0.5%) for all breast cancer patients tested (1.31% of the 76 patients aged 40 years or less, or 7.7% of the 13 patients aged 30 years or less). Of the ovarian DNA samples analysed, 0/80 (0%) were found to contain the *BRCA1* 5382insC mutation.

The RG-PCR result for the positive breast cancer sample (BC185), along with appropriate negative and positive control samples, is shown in the Figure. The single-strand conformational polymorphis (SSCP) band patterns observed for this sample were identical to those seen in the positive sample from the West Lothian 5382insC kindred, but different from those observed in normal individuals (data not shown).

### Haplotype analysis

Haplotype analysis of the single sample found to be positive for the 5382insC mutation was performed using the markers D17S855, D17S1322, D17S1323 and D17S1327. Using the convention for describing allele sizes established by Simard et al (1994), our positive sample had the haplotype D/A for D17S855, E/E for D17S1322, F/F for D17S1323 and O/M for D17S1327. Haplotyping carried out with five affected members of the West Lothian kindred along with BC185 revealed a 2-bp difference between BC185 (DEFO) and all five kindred members (DEFN). Since the haplotype commonly found with the 5382insC mutation is DEFO, the genotype for our positive sample would be consistent with this.

### DISCUSSION

The initial hypothesis that 'given the existence of a very large kindred with the *BRCA1* 5382insC mutation in the West Lothian region, the incidence of this mutation might be considerably higher for women with breast cancer within a Scottish population' was shown to be unfounded. However, the identification of the 5382insC mutation in a 26-year-old woman from our cohort of patients with breast cancer highlights a low, but significant, incidence of the *BRCA1* 5382insC mutation. Indeed, we believe this represents the only reported incidence of the mutation in a sporadic population of breast cancer patients. While only 13 breast cancer patients aged 30 years or less were available for study, the single sample having the mutation in question was shown to belong to this group (representing a mutation frequency of almost 8% within this subpopulation).

Haplotyping of the sporadic breast cancer, BC185, along with members of the Scottish West Lothian kinship, revealed the presence of a single telomeric difference in the haplotype commonly associated with the 5382insC mutation in the West Lothian kinship. Interestingly, it is the West Lothian kindred that is unusual, since the haplotype associated with the 5382insC mutation differs at the marker D17S1327, being 2 bp larger (DEFN) than the common haplotype (DEFO) reported previously. Despite this unexpected finding, particularly in view of the common haplotype found in the sporadic case BC185, this observation has been reported previously by Gayther et al (1995) (family BO82), and this was subsequently confirmed for this study. This recombination strengthens the suggestion that this mutant chromosome is a relatively old mutation.

In conclusion, these results suggest that the Scottish mutant haplotypes, although not identical, are likely to be derived from the same ancestral chromosome as those found in Canada. Furthermore, we believe that all Scottish women from suspected breast cancer families should be screened for the mutation 5382insC, particularly in the light of the frequency detection rate found in this population of sporadic breast cancers.

### ACKNOWLEDGEMENTS

The authors would like to express their thanks to Mr M Dixon and Mr U Chetty for the collection of breast tumour material and to Miss June Telford for making samples available.

### REFERENCES

Castilla LH, Couch FJ, Erdos MR, Hoskins KF, Calzone K, Garber JE, Boyd J Lubin MB, Deshano ML, Brody LC, Collins FS and Weber BL (1994) Mutations in the *BRCA1* gene in families with early onset breast and ovarian cancer. *Nature Genet* **8**: 387–391

Claus EB, Risch N and Thompson WD (1991) Genetic analysis of breast cancer in the cancer and steroid hormone study. *Am J Hum Genet* **48**: 232–241

Easton DF, Bishop DT, Ford D, Cockford GP and the Breast Cancer Linkage Consortium (1993) Genetic linkage analysis in familial breast and ovarian cancer. *Am J Hum Genet* **52**: 718–722

Friedman LS, Ostermeyer EA, Szabo CI, Dowd P, Lynch ED, Rowell SE and King M-C (1994) Confirmation of *BRCA1* by analysis of germline mutations linked to breast and ovarian cancer in ten families. *Nature Genet* **8**: 399–404

Futreal PA, Liu Q, Shattuck-Eidens D, Cochran C, Harshman K, Tavtigian S, Bennett LM, Haugen-Shano A, Swensen J, Miki Y, Eddington K, McCure M, Frye C, Weaver-Feldhaus J, Ding W, Gholami Z, Soderkvist P, Terri L, Jhanwar S, Berchunk A, Iglehart JD, Marks J, Ballinger DG, Barrett JC, Slilnick MH, Kamb A and Wiseman R (1994) *BRCA1* mutations in primary breast and ovarian carcinomas. *Science* **266**: 120–122

Gayther SA, Warren W, Mazoyer S, Russell PA, Harrington PA, Chiano M, Seal S, Hamoudi R, van Rensburg EJ, Dunning AM, Love R, Evans G, EAston D, Clayton D, STratton MR and Ponder BAJ (1995) Germline mutations of the *BRCA1* gene in breast and ovarian cancer families provide evidence for a genotype–phenotype correlation. *Nature Genet* **11**: 428–433

Goldgar DE, Fields P, Lewis CM, Tran TD, Cannon-Albright LA, Ward JH, Swensen J and Skolnick MH (1994) A large kindred with 17q-linked breast and ovarian cancer; genetic, phenotypic and genealogic analysis. *J Natl Cancer Inst* **86**: 200–209

Hall JM, Lee M-K, Newman B, Morrow JE, Anderson LE, Huey B and King M-C (1990) Linkage analysis of early onset familial breast cancer to chromosome 17q21. *Science* **250**: 1684–1689

Miki Y, Swensen J, Shattuck-Eidens D, Futreal PA, Harshman K, Tavtigian S, Liu Q, Cochran C, Bennet LM, Ding W, Bell R, Rosenthal J, Hussey C, Tran T, McLure M, Frye C, Hattier T, Phelps R, Haugen-strano A, Katcher H, Yakumo K, Gholami Z, Shaffer D, Stone S, Bayer S, Wray C, Bodgen R, Dayananth P, Ward J, Tonin P, Narod S, Bristow PK, Norris FH, Helvering L, Morrison P, Rosteck P, Lai, M, Barrett JC, Lewis C, Neuhausen S, CannonAlbright L, Goldgar D, Wiseman R, Kamb A and Skolnick (1994) A strong candidate for the breast and ovarian cancer susceptibility gene *BRCA1*. *Science* **266**: 66–71

Narod SA, Feunteun J, Lynch HT, Watson P, Conway T, Lynch J and Lenoir GM (1991) Familial breast–ovarian cancer locus on chromosome 17q12–23. *Lancet* **338**: 82–83

Narod SA, Ford D, Devilee P, Barkardottir RB, Lynch HT, Smith SA, Ponder BAJ, Weber BL, Garber JE, Birch JM, Cornelis RS, Kelsell DP, Spurr NK, Smyth E, Haites N, Sobol H, Bignon Y-J, Chang-Claude J, Hamann U, Lindblom A, Borg A, Piver MS, Gallion HH, Struewing JP, Whittemore A, Tonin P, Goldgar DE, EAston DF, and the Breast Cancer Linkage Consortium (1995) An evaluation of genetic heterogeneity in 145 breast–ovarian cancer families. *Am J Hum Genet* **56**: 254–264

Neuhausen SL, Mazoyer S, Friedmand L, Stratton M, Offit K, Caligo A, Tomlinson G, Cannon-Albright L, Bishop T, Kelsell D, Solomon E, Weber B, Couch F, Struewing J, Tonin P, Durocher F, Norod, S, Skolnick MH, Lenoir G, Serova O, Ponder B, Stoppa-Lyonnet D, Easton D, Kink M-C and Golgar DE (1995) A P1

© Cancer Research Campaign 1997 

based physical map of the region from D17S766 to D17S78 containing the breast cancer susceptibility gene BRCA1. *Hum Mol Genet* **3**: 1919–1926

Newman B, Austin MA, Lee M and King M-C (1988) Inheritance of breast cancer: evidence for autosomal dominant transmission in high risk families. *Proc Natl Acad Sci USA* **85**: 1–5

Simard J, Tonin P, Durocher F, Morgan K, Rommens J, Gingras S, Samson C, Leblanc J-F, Belanger C, Dion F, Liu Q, Skolnick M, Goldgar D, Shattuck-Eidens D, Labrie F and Narod SA (1994) Common origins of BRCA1 mutations in Canadian breast and ovarian cancer families. *Nature Genet* **8**: 392-398

Warner J, Barron L, Goudie D, Kelly K, Dow D, Fitzpatrick DR and Brock DJH (1996) A general method for the detection of CAG repeat expansions by fluorescent PCR. *J Med Genet* **33**: 1022–1026

Weissenbach J, Grape G, Dib C, Vignal A, Morissette P, Vaysseix G and Lathrop M (1992) A second generation linkage map of the human genome. *Nature* **359**: 794–801

 © Cancer Research Campaign 1997